# The Effects of One-Anastomosis Gastric Bypass on Fatty Acids in the Serum of Patients with Morbid Obesity

**DOI:** 10.1007/s11695-021-05531-6

**Published:** 2021-07-13

**Authors:** Alicja Pakiet, Łukasz P Haliński, Olga Rostkowska, Łukasz Kaska, Monika Proczko-Stepaniak, Tomasz Śledziński, Adriana Mika

**Affiliations:** 1 Department of Environmental Analysis, Faculty of Chemistry, University of Gdańsk, Wita Stwosza 63, 80-309 Gdańsk, Poland; 2 Department of General, Endocrine and Transplant Surgery, Faculty of Medicine, Medical University of Gdańsk, Smoluchowskiego 17, 80-214 Gdańsk, Poland; 3 Department of Pharmaceutical Biochemistry, Faculty of Pharmacy, Medical University of Gdańsk, Debinki 1, 80-211 Gdańsk, Poland

**Keywords:** Morbid obesity, Bariatric surgery, One-anastomosis gastric bypass, Fatty acid

## Abstract

**Purpose:**

Obesity is associated with alterations in serum fatty acid profiles. One-anastomosis gastric bypass is a type of bariatric surgery used in the treatment of morbid obesity. The aim of this study was to establish if, between 6 and 9 months after this procedure, the fatty acid composition in the serum of patients normalizes to values similar to the healthy, lean population.

**Materials/Methods:**

The study included 46 patients that underwent surgical treatment for obesity with one-anastomosis gastric bypass. The serum fatty acid composition was determined using gas chromatography-mass spectrometry. Principal component analysis was conducted to detect the differences between fatty acid profiles in patients pre- and post-surgery, and in 29 control nonobese subjects.

**Results:**

Patients with morbid obesity were characterized by lowered levels of beneficial odd- and branched-chain fatty acids and polyunsaturated fatty acids. While the odd- and branched-chain fatty acid amounts normalized 6–9 months after bariatric treatment, the polyunsaturated fatty acid levels did not. Moreover, the total fatty acid profiles of patients pre- and post-bariatric surgery were still markedly different than those of lean, healthy controls.

**Conclusion:**

Following one-anastomosis gastric bypass, there are some beneficial changes in serum fatty acids in treated patients, possibly due to weight loss and dietary regimen changes. However, they may be insufficient to restore the proper levels of other fatty acids, which may need to be additionally supplemented.

**Graphical abstract:**

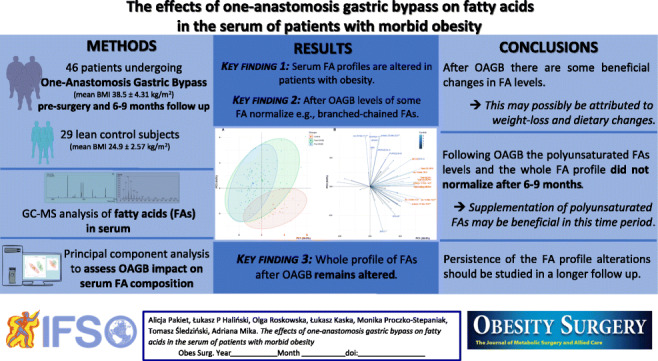

## Introduction

The myriad severe health impacts of obesity prompt the need for effective treatment. Usually, obesity is treated by encouraging lifestyle changes that promote healthy activity levels and a suitable diet [1]. However, the most effective method of morbid obesity treatment is bariatric surgery [2]. Among available surgical methods, the most regularly performed are laparoscopic sleeve gastrectomy (LSG), Roux-en-Y gastric bypass (RYGB), gastric banding, or biliopancreatic diversion [3]. Besides weight loss, bariatric treatment can be effective in addressing obesity comorbidities, for example, the remission of type II diabetes mellitus (T2DM) [3, 4], dyslipidaemia, or hypertension [4]. One-anastomosis gastric bypass (OAGB), previously known as single-anastomosis gastric bypass or mini gastric bypass, is one of the newest, malabsorptive bariatric procedures [5]. Alongside excellent weight loss outcomes [6, 7], OAGB provides a favourable degree of remission of comorbidities, e.g. T2DM, arterial hypertension, joint disease, or sleep apnoea [7]. An improvement in dyslipidaemia following bariatric treatment is also confirmed in the studies on OAGB [8, 9]. However, limiting the investigation of lipid changes to cholesterol and lipoprotein profiles leaves out a multitude of data. Fatty acids (FAs) are primary components of almost all lipid species, and their composition, arising as the sum of endogenous synthesis and dietary intake, can provide valuable information when we consider different, sometimes even opposing effects of various FA groups on metabolism [10]. OAGB, as every bariatric procedure, may influence the FA intake and absorption from food, but also may change their metabolism in patients’ bodies. Since some groups of FAs are considered beneficial, e.g. n-3 polyunsaturated FAs (PUFAs), whereas others are adverse to human health, e.g. saturated FAs (SFAs) [10], every change in their levels may have an important effect on the metabolic outcome of a bariatric surgery. Recently, in our work, we showed that OAGB reconstitutes the appropriate serum amino acid profiles to the levels measured in nonobese, healthy people [9]. The primary aim of this study is to verify if OAGB also reconstitutes the appropriate serum fatty acid profiles to the levels similar to lean, healthy people. The secondary aim is to examine the changes in individual FAs after surgery.

## Materials and Methods

### Study Subjects

The study included 46 patients (7 male, 39 female) with morbid obesity that underwent surgical treatment with OAGB at the Department of General, Endocrine and Transplant Surgery, at the Medical University of Gdańsk, between 2016 and 2018. The subjects included 23 patients suffering from T2DM and the remaining 23 had reference glucose levels. The patients on lipid-lowering drugs were excluded from the study. Twenty-nine lean individuals (17 male, 12 female) without metabolic disorders made up the control group. Blood samples were drawn after an overnight fast, and serum was obtained after centrifugation and stored at – 80 °C until analysis. Anthropometric and laboratory parameters were measured at baseline (before surgery) in the OAGB patients and the control group, and again 6–9 months after the surgery in the OAGB patients, the results of which are collected in Table 1. Routine laboratory parameters were determined at the Central Clinical Laboratory, at the Medical University of Gdańsk.

**Table 1.** Comparison of metabolic characteristics between patients with morbid obesity before and after OAGB, and lean controls

	Control	Pre-OAGB	Post-OAGB	*p* (control vs pre-OAGB)	*p* (pre- vs post-OAGB)	*p* (control vs post-OAGB)
Age	49.7 ± 11.3	48.6 ± 10.6		0.685	-	0.685
BMI (kg/m^2^)	24.9 ± 2.57	38.5 ± 4.31	29.6 ± 3.85	< 0.001	< 0.001	< 0.001
HbA1C (%)	-	5.79 ± 0.88	5.24 ± 0.47	-	0.003	-
TG (mg/dL)	109 ± 47.7	113 ± 37.3	87.8 ± 26.7	0.772	0.006	0.080
HDL (mg/dL)	55.3 ± 13.2	50.1 ± 9.33	50.9 ± 11.7	0.125	0.653	0.273
LDL (mg/dL)	128 ± 41.7	114 ± 33.8	88.3 ± 25.3	0.241	0.027	< 0.001
TC (mg/dL)	208 ± 44.5	201 ± 40.9	180 ± 49.9	0.486	0.018	0.039
CRP (mg/L)	1.57 ± 1.22	1.65 ± 0.53	1.02 ± 0.55	0.769	0.011	0.075
Albumin (g/L)	40.0 ± 2.34	37.4 ± 7.60	37.1 ± 2.45	0.135	0.832	< 0.001
Creatinine (mg/dL)	0.86 ± 0.16	0.80 ± 0.22	0.71 ± 0.17	0.246	< 0.001	< 0.001
Glucose (mg/dL)	93.1 ± 9.36	111 ± 32.1	91.5 ± 11.2	0.004	< 0.001	0.468
Insulin (μU/mL)	9.11 ± 3.97	14.9 ± 7.85	7.72 ± 6.49	0.002	< 0.001	0.335
HOMA-IR	2.13 ± 1.02	4.37 ± 3.04	2.04 ± 1.96	0.001	< 0.001	0.804

Values are mean ± SD. Abbreviations: *BMI*, body mass index; *CRP*, C-reactive protein; *HbA1C*, glycated haemoglobin; *HDL*, high-density lipoprotein cholesterol; *HOMA*, homeostatic model assessment of insulin resistance; *LC*, lean controls; *LDL*, low-density lipoprotein cholesterol; *TG*, triacylglycerols; *TC*, total cholesterol. These data were also presented in our previous study performed on the same group of subjects [9]

The OAGB has been designed following the procedure described by García-Caballero et al. [11]. The gastric pouch was calibrated using the 36F bougie. The 2.5-cm side to side pouch-jejunum anastomosis was created using linear GIA staplers. To reduce the risk of biliary reflux, the suspension suture fixing the proximal (to anastomosis) part of the jejunum to the bypassed stomach has been done as well as derotation fixation of the distal (to anastomosis) part of jejunum. In the BMI range of the collected patients, the excluded jejunum limb from the alimentary transit has been calculated for 150–200 cm. To count the length of the excluded intestine, we use Storz surgical tools with a 10-cm line marked on the shaft—so we can use the tool as a ruler and measure the desired length. Our bariatric centre is certified in accordance with the requirements of IFSO (IFSO Centre of Excellence) [12]. The study was conducted in compliance with the Declaration of Helsinki of the World Medical Association, and the protocol was approved by the Local Bioethics Committee at the Medical University of Gdańsk (approval no. NKBBN/493/2016). Written, informed consent was obtained from all participants prior to the study.

After the surgery, the patients were advised to adhere to a low-calorie diet (maximum of 1200 kcal for women, and 1500 kcal for men) with a macronutrient composition as follows: 25–30% protein, up to 30% fats, and the remaining 35–40% carbohydrates. The recommended dietary fat sources were fish and poultry, dairy products, e.g. cottage cheese, low-fat yoghurt, and mozzarella cheese; vegetable fats—olive oil or rapeseed oil; and seeds, e.g. sesame, flax, pumpkin, and nuts. Sweets were forbidden. The patients did not take an omega fatty acid supplement.

### GC-MS Analysis of Fatty Acids

Total lipids were extracted from serum aliquots with a mixture of chloroform-methanol (2:1, v/v), as described by Folch et al. [13]. The chloroform phase was collected, dried under a nitrogen stream, and hydrolyzed with 1 mL of 0.5 M KOH in methanol at 90 °C. After 3 h of incubation, the mixture was acidified with 6 M HCl, 1 mL H_2_O was added, and free FAs were extracted thrice with 1 mL of *n*-hexane. Fatty acid methyl esters (FAMEs) were prepared by derivatization with 10% BF_3_-methanol solution (55 °C, 1.5 h). Water was added to the mixture, and FAMEs were extracted thrice with *n*-hexane, as described above. The samples were dried under a nitrogen stream and stored at – 20 °C until analysis.

The analysis of FAMEs was conducted using a GC-EI-MS QP-2010SE spectrometer (Shimadzu, Kyoto, Japan) on a Zebron ZB-5MSi capillary column, 30 m × 0.25 mm i.d. × 0.25-μm film thickness (Phenomenex, Torrance, CA, USA). The analysis run time was 60 min with the column oven set at 60–300 °C (4 °C/min). The carrier gas was helium with the column head pressure at 100 kPa. The electron impact source for mass spectrometry detection operated at 70 eV, with the mass scan range set at m/z 45–700 in the full scan mode. The identification of FAs was aided by the use of reference standards (37 FAME Mix, Sigma Aldrich, St. Louis, MO, USA), and the reference library NIST 11. 19-methylarachidic served as an internal standard.

### Data Analysis

Principal component analysis (PCA) was carried out in the R computing environment [14] using the FactoMineR package [15], with data visualization done with the factoextra package. The data for analysis were auto scaled. The PCA results were subjected to one-way analysis of variance (ANOVA) with the post hoc Tukey-Kramer test for data with normal distribution. A nominal p-value < 0.01 was considered significant for PCA. Additionally, comparisons between two study groups at a time were carried out with the independent samples two-tailed *t*-test (between the control and patient groups) or the paired two-tailed *t*-test (between pre- and post-OAGB patients). A nominal p-value < 0.05 was considered significant for the *t-*tests. The statistical analysis was performed in SigmaPlot (Systat Software Inc., San Jose, CA, USA) and Gnumeric (GNOME Foundation, Orinda, CA, USA).

## Results

Patients with morbid obesity after OAGB exhibited a significant decrease in body mass index and fasting blood glucose concentration as well as an improvement in the levels of triglycerides, and total and LDL-cholesterol (Table 1). We did not detect any impact of OAGB on HDL-cholesterol levels.

**Figure 1.**
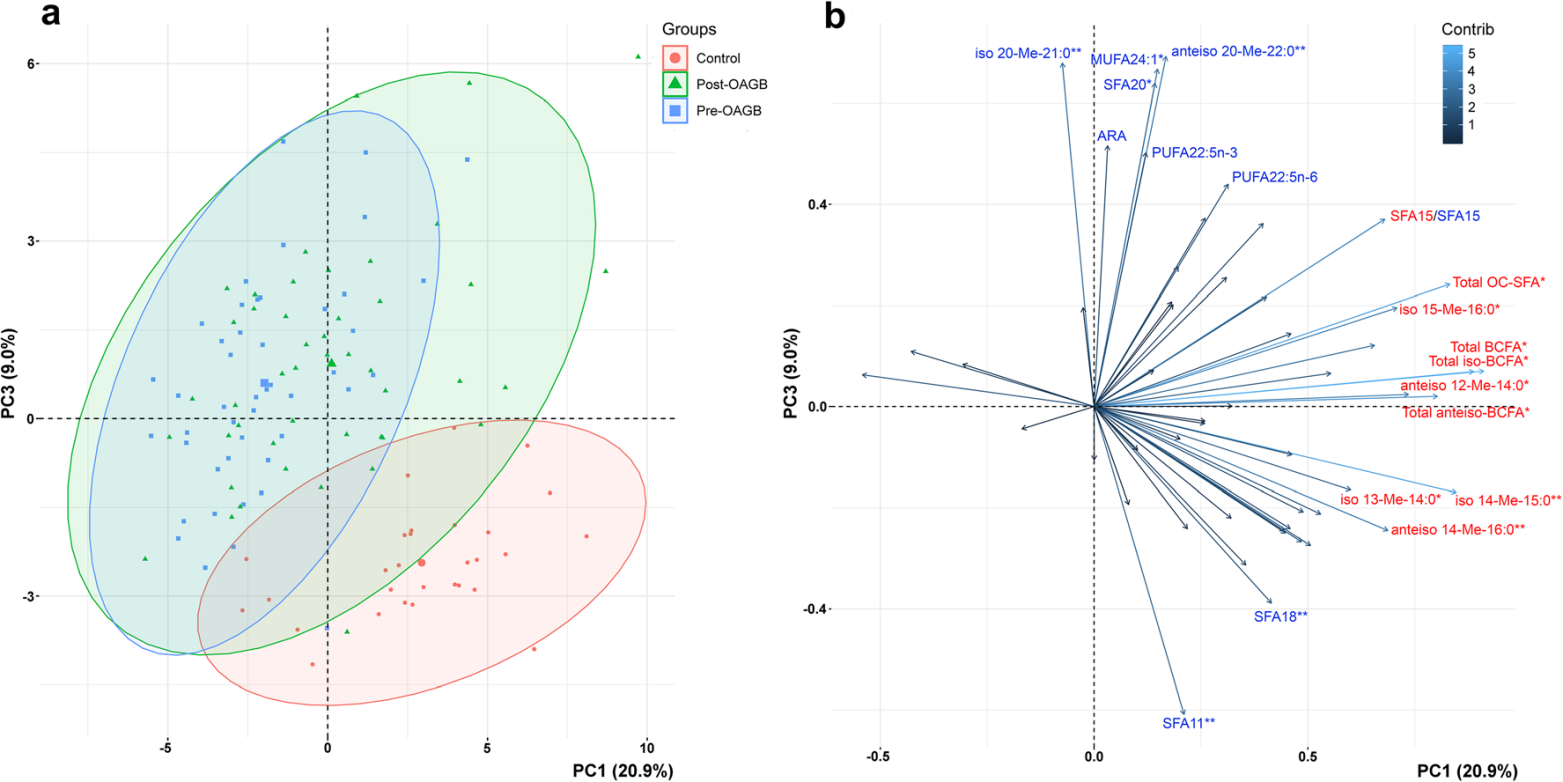
PCA results of whole FA profiles in the study subjects. Score plot (**a**) and variable plot (**b**) for the first and third PCs. *—variables that differ significantly between one control-OAGB pair (control and pre-OAGB or control and post-OAGB), **—variables that differ between the control and both the pre- and post-OAGB group, the p-value was set as significant at p < 0.01. Red—FAs with the most significant contribution to PC1, blue—FAs with the most significant contribution to PC3

**Figure 2.**
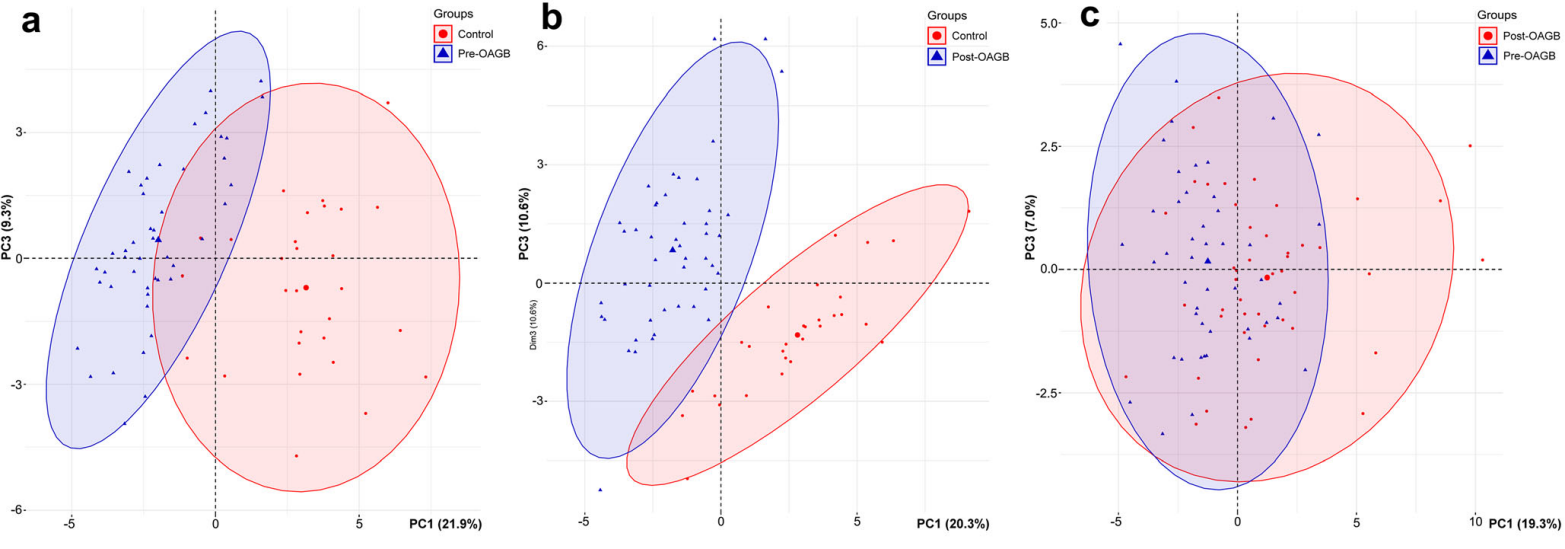
PCA results of individual pairs of cases based on single FA profiles. Score plot for the first and third PCs. **a** Control group vs pre-OAGB. **b** Control vs post-OAGB. **c** Post-OAGB vs pre-OAGB. Contribution of single FAs to each principal component was similar to the one presented in Figure 1. Partial separation of control group from **a** pre-OAGB and **b** post-OAGB patients is visible because of the different PC1 values in each group. **c** No separation was obtained for patients before and after the surgery

A PCA plot based on the analysis of whole FA profiles revealed a grouping of pre- and post-OAGB patients and a slight separation of the control group (Figure 1). PC1 was responsible for 20.9% of the total variance and accounted for the partial separation of the lean controls from the bariatric patients, both pre- and post-OAGB, based on statistically significant lower levels of iso-20-M-21:0, 24:1, and 20:0 and anteiso-20-M-22:0, and higher levels of 11:0 and 20:0. Additionally, some PUFAs were contributing factors in PC1; however, these differences were not statistically significant. An overview of the mean PC1-3 values obtained for each group is given in Table 2. We then considered control-OAGB patient pairs separately based on the individual FA profiles exclusively (Figure 2), which additionally confirmed the tendency of the control group to separate from both the pre- (Figure 2a, PC1 21.9%, PC3 9.3%) and post-OAGB (Figure 2b, PC1 20.3%, PC3 10.6%) patients. The separation between the pre- and post-OAGB patients was better, although not complete (Figure 2c, PC1 19.3%, PC3 7.0%).

**Table 2.** Average values of principal components 1–3 from the PCA based on whole FA profiles, obtained for individuals from the healthy control, pre-OAGB, and post-OAGB groups

PC no.	Control	Pre-OAGB	Post-OAGB
1	2.94 ± 2.71a	− 1.98 ± 2.16b	0.12 ± 3.26c
2	− 1.65 ± 2.80a	− 0.39 ± 2.29a	1.44 ± 2.94b
3	− 2.43 ± 0.93a	0.60 ± 1.82b	0.93 ± 1.95b

Values are mean ± SD. Values in a single row not sharing the same letter are significantly different from each other (Tukey-Kramer, p < 0.01)

A comparison of individual FAs between study groups is presented in Table 3. A total of 44 FAs were identified. In subjects with morbid obesity before surgery, when compared to lean controls, we observed lowered total odd-chain FAs (OCFAs), iso- and anteiso-branched-chain FAs (BCFAs), and n-3 and n-6 PUFA content. Additionally, in patients with morbid obesity, we observed lover levels of eicosapentaenoic acid (EPA) and essential PUFAs of both n-3 series—α-linolenic acid (ALA, p < 0.001)—and of n-6 series—linoleic acid (LA, p < 0.001). Monounsaturated FA (MUFA) content was elevated, which was caused mainly by higher oleic acid (18:1) levels. In the follow-up after the OAGB procedure, there was an improvement in the BCFA content, which was then not significantly different to that observed in lean controls. We also observed a moderate, insignificant increase in OCFA levels after OAGB; however, the levels of OCFAs after bariatric surgery were slightly lower, but not significantly different to the control levels. After the procedure, we did not notice an improvement in PUFA levels. The n-3 series PUFAs of post-OAGB patients were even lower than those of the pre-OAGB group (p = 0.030). Moreover, the MUFAs, which are incorporated into triglycerides, were even higher in post-OAGB patients, mainly as a result of the higher oleic acid content.

**Table 3.** Serum FA composition (%) in the study subjects

Fatty acid	Control	Pre-OAGB	Post-OAGB	p (control vs pre-OAGB)	p (pre- vs post-OAGB)	p (control vs post-OAGB)
10:0	0.021 ± 0.001	0.027 ± 0.006	0.009 ± 0.001	0.278	0.003	< 0.001
12:0	0.223 ± 0.018	0.090 ± 0.006	0.157 ± 0.018	< 0.001	< 0.001	0.009
14:0	1.16 ± 0.057	0.78 ± 0.044	1.13 ± 0.067	< 0.001	< 0.001	0.678
16:0	23.0 ± 0.347	25.0 ± 0.255	24.6 ± 0.272	< 0.001	0.084	0.001
18:0	7.20 ± 0.137	6.26 ± 0.087	6.45 ± 0.097	< 0.001	0.016	< 0.001
20:0	0.078 ± 0.004	0.092 ± 0.003	0.090 ± 0.004	0.013	0.762	0.029
22:0	0.151 ± 0.010	0.158 ± 0.006	0.125 ± 0.004	0.574	< 0.001	0.019
24:0	0.142 ± 0.008	0.120 ± 0.005	0.096 ± 0.005	0.017	< 0.001	< 0.001
*ECFA*	*32.0 ± 0.350*	*32.5 ± 0.261*	*32.6 ± 0.292*	*0.263*	*0.733*	*0.205*
11:0	0.014 ± 0.001	0.006 ± 0.001	0.005 ± < 0.001	< 0.001	0.085	< 0.001
13:0	0.028 ± 0.002	0.015 ± 0.001	0.015 ± 0.001	< 0.001	0.679	< 0.001
15:0	0.237 ± 0.010	0.228 ± 0.008	0.268 ± 0.012	0.493	< 0.001	0.060
17:0	0.259 ± 0.007	0.239 ± 0.006	0.247 ± 0.008	0.034	0.201	0.278
19:0	0.034 ± 0.003	0.019 ± 0.001	0.021 ± 0.001	< 0.001	0.473	< 0.001
21:0	0.015 ± 0.002	0.013 ± 0.001	0.011 ± 0.001	0.274	0.307	0.081
23:0	0.057 ± 0.004	0.057 ± 0.003	0.038 ± 0.002	0.968	< 0.001	< 0.001
*OCFA*	*0.644 ± 0.020*	*0.577 ± 0.013*	*0.606 ± 0.019*	*0.008*	*0.075*	*0.184*
4,8,12-M-13:0	0.011 ± 0.001	0.010 ± 0.001	0.014 ± 0.002	0.680	0.050	0.162
*Anteiso* 12-M-14:0	0.045 ± 0.003	0.027 ± 0.002	0.043 ± 0.003	< 0.001	< 0.001	0.562
*Anteiso* 14-M-16:0	0.120 ± 0.007	0.065 ± 0.004	0.093 ± 0.006	< 0.001	< 0.001	0.005
*Anteiso* 16-M-18:0	0.030 ± 0.002	0.030 ± 0.002	0.040 ± 0.002	0.749	< 0.001	< 0.001
*Anteiso* 20-M-22:0	0.008 ± 0.001	0.013 ± 0.001	0.015 ± 0.001	< 0.001	0.063	< 0.001
*Anteiso BCFA*	*0.203 ± 0.009*	*0.132 ± 0.006*	*0.191 ± 0.009*	*< 0.001*	*< 0.001*	*0.343*
*Iso* 12-M-13:0	0.009 ± 0.001	0.008 ± 0.001	0.008 ± 0.001	0.497	0.575	0.191
*Iso* 13-M-14:0	0.032 ± 0.002	0.022 ± 0.002	0.027 ± 0.002	0.001	0.018	0.070
*Iso* 14-M-15:0	0.075 ± 0.003	0.040 ± 0.002	0.056 ± 0.003	< 0.001	< 0.001	< 0.001
*Iso* 15-M-16:0	0.089 ± 0.006	0.072 ± 0.004	0.090 ± 0.005	0.015	< 0.001	0.885
*Iso* 20-M-21:0	0.003 ± < 0.001	0.009 ± 0.001	0.010 ± 0.001	< 0.001	0.032	< 0.001
*iso BCFA*	*0.208 ± 0.010*	*0.151 ± 0.006*	*0.191 ± 0.008*	*< 0.001*	*< 0.001*	*0.185*
*Total BCFA*	*0.422 ± 0.017*	*0.295 ± 0.011*	*0.396 ± 0.017*	*< 0.001*	*< 0.001*	*0.270*
CPOA2H	0.164 ± 0.006	0.155 ± 0.005	0.163 ± 0.009	0.213	0.307	0.862
*Total SFA*	*33.1 ± 0.343*	*33.4 ± 0.264*	*33.6 ± 0.302*	*0.491*	*0.418*	*0.259*
14:1	0.072 ± 0.006	0.042 ± 0.003	0.065 ± 0.007	< 0.001	< 0.001	0.498
16:1	2.84 ± 0.146	2.99 ± 0.112	3.34 ± 0.137	0.400	0.002	0.013
18:1	26.5 ± 0.576	29.8 ± 0.369	30.2 ± 0.398	< 0.001	0.290	< 0.001
19:1	0.027 ± 0.002	0.023 ± 0.001	0.025 ± 0.002	0.149	0.329	0.496
20:1	0.175 ± 0.006	0.158 ± 0.006	0.139 ± 0.004	0.046	0.002	< 0.001
22:1	0.035 ± 0.005	0.016 ± 0.001	0.017 ± 0.001	< 0.001	0.645	0.001
24:1	0.232 ± 0.020	0.257 ± 0.015	0.332 ± 0.014	0.317	< 0.001	< 0.001
*MUFA*	*29.9 ± 0.647*	*33.4 ± 0.392*	*34.3 ± 0.462*	*< 0.001*	*0.056*	*< 0.001*
18:3 n-3 (ALA)	0.323 ± 0.016	0.201 ± 0.014	0.218 ± 0.012	< 0.001	0.203	< 0.001
20:5 n-3 (EPA)	1.09 ± 0.152	0.69 ± 0.049	0.62 ± 0.035	0.017	0.190	0.005
20:4 n-3	0.100 ± 0.006	0.050 ± 0.003	0.042 ± 0.003	< 0.001	0.002	< 0.001
22:6 n-3 (DHA)	1.17 ± 0.087	1.25 ± 0.058	1.09 ± 0.047	0.466	0.001	0.399
22:5 n-3 (DPA n-3)	0.286 ± 0.010	0.296 ± 0.011	0.329 ± 0.013	0.537	0.001	0.013
*PUFA n-3*	*2.97 ± 0.238*	*2.49 ± 0.102*	*2.30 ± 0.079*	*0.068*	*0.030*	*0.011*
18:2 n-6 (LA)	26.9 ± 0.668	23.2 ± 0.525	22.7 ± 0.548	< 0.001	0.284	< 0.001
22:2 n-6 (ARA)	5.53 ± 0.219	6.17 ± 0.268	5.62 ± 0.188	0.069	0.008	0.745
20:3 n-6 (DGLA)	1.13 ± 0.046	1.03 ± 0.035	1.13 ± 0.037	0.067	0.008	0.923
20:2 n-6	0.167 ± 0.006	0.123 ± 0.005	0.148 ± 0.004	< 0.001	< 0.001	0.016
22:5 n-6 (DPA n-6)	0.061 ± 0.005	0.056 ± 0.007	0.060 ± 0.005	0.570	0.367	0.948
22:4 n-6 (AdA)	0.099 ± 0.006	0.103 ± 0.004	0.134 ± 0.023	0.582	0.192	0.143
*PUFA n-6*	*33.9 ± 0.686*	*30.7 ± 0.539*	*29.8 ± 0.612*	*< 0.001*	*0.096*	*< 0.001*

Values are mean ± SD. Abbreviations: *AdA*, adrenic acid; *ALA*, α-linolenic acid; *ARA*, arachidonic acid; *BCFA*, branched-chain FA; *CPOA2H*, cyclopropaneoctanoic acid 2-hexyl; *DGLA*, dihomo-γ-linolenic acid; *DHA*, docosahexaenoic acid; *DPA*, docosapentaenoic acid; *ECFA*, even-chain FA; *EPA*, eicosapentaenoic acid; *LA*, linoleic acid; *MUFA*, monounsaturated FA; *OCFA*, odd-chain FA; *PUFA*, polyunsaturated FA; *SFA*, saturated FA; Italics - major groups of fatty acid

## Discussion

Our study aimed to evaluate whether OAGB-induced weight loss leads to the normalization of FA profiles to the levels observed in the healthy, lean population. Despite improvements in the composition of some pivotal bioactive FAs, the deregulation of FA profiles associated with obesity seems to persist in patients following OAGB treatment for 6–9 months after the surgery, as evidenced by the grouping of pre- and post-OAGB groups (Figure 1). This observation was dissimilar to the trend to normalization that we previously reported for serum amino acids [9]. We did, however, observe significant changes in some individual FAs or groups of FAs.

An important group of FAs explored in our study are BCFAs, a group primarily derived from dairy consumption, albeit possibly arising from the catabolism of branched-chain amino acids in visceral adipose tissue [16]. Recent evidence suggests that BCFAs have a positive influence on inflammation, energy, and glucose homeostasis, and possess anti-cancer properties [17]. Previously, we reported that OAGB-induced weight loss is associated with an increase in serum BCFAs [16] after an initial, short-term decrease following the surgery [18]. This study reinforces the finding that serum BCFA levels normalize 6–9 months after the surgery. OCFAs, a group of FAs found in dairy products, have been shown to be inversely associated with the incidence of coronary heart disease and T2DM risk [19]. Jenkins et al. [20] postulated a strong relationship between the endogenous synthesis of heptanoic acid 17:0 and glucose intolerance. Our results showed an insignificant trend of an increase in OCFA levels after OAGB. However, contrary to OCFA levels in patients before surgery, the levels of these FAs after surgery did not differ significantly from nonobese subjects. This suggests a partial normalization of OCFAs after OAGB, which may contribute to the improvement of glucose homeostasis and the reduction of CVD risk after OAGB.

Contrary to other studies [21, 22], we did not find significant differences between the amount of SFAs in serum after bariatric surgery, suggesting that OAGB-induced weight loss does not influence this group. The proportion of MUFAs in patients with obesity was significantly higher than in lean controls and this persisted after OAGB. The primary contributor to this increase was oleic acid, the blood content of which is reflective of dietary habits, as indicated by controlled feeding studies [23]. Moreover, in the period following a bariatric surgery, patients experience continuous weight loss [6], wherein the FAs in existing adipose tissue depots are mobilized. FAs abundant in adipose tissues, i.e. saturated 16:0 and 18:0, and especially oleic acid, the most abundant in adipose tissue, which is the major component of triacylglycerols [23, 24], are probably released in the highest amounts. This may be one of the reasons for the increased level of oleic acid in the blood.

At baseline, patients with obesity exhibited significantly lowered n-3 and n-6 PUFA content. Six–nine months after OAGB, we did not observe an increase in total PUFA content to the levels observed in healthy subjects. The total levels remained lower than in the control group for n-6 series PUFAs, or were even further reduced in the case of n-3 PUFAs (8% decrease from the preoperative status at the significance p = 0.030). Previously, we reported a short-term (after 2 weeks) decrease in these FAs in the serum of post-OAGB patients [18]. This decrease was then more pronounced than it is after a longer follow-up, suggesting that the levels of these FAs tend to normalize slowly and may restore to those of the lean population after more time has passed. However, the decreased n-3 PUFA in 6–9 months after OAGB may be detrimental for patients after bariatric surgery, and suggests the need for the supplementation of this group of FAs. Due to the anti-inflammatory, cardioprotective, and anti-cancer properties of n-3 PUFA [25], their decreased levels after OAGB may increase the risk of inflammation, cardiovascular diseases, and cancers, all of which are associated with obesity. Lin et al. [26] reported a lasting decrease of ALA and EPA serum levels in the first year after biliopancreatic diversion with a duodenal switch (BPDDS) surgery, and a trend towards an increase in EPA levels following LSG, while Forbes et al. [27] in a 6-month follow-up after RYGB observed a persistent decrease in EPA concentrations in plasma phospholipids. In our study, the levels of these FAs returned to preoperative levels in the 6–9-month follow-up period, after the initial decrease we reported previously [18]. Since the storage of n-3 PUFAs in adipose tissue is limited [28], the observed changes are probably due to reduced dietary intake after bariatric intervention. However, it should be noted that despite the fact that our patients were not additionally supplementing n-3 PUFAs, the amounts of ALA and EPA serum were not significantly different pre- and post-OAGB, in contrast to studies of other bariatric procedures, where the amounts of these FAs after the bariatric procedures were even lower [26, 27]. We can only speculate that the lack of decrease of ALA and EPA after OAGB may be a beneficial effect of this type of bariatric surgery compared to DS and RYGB, but it may also be an effect of the differences in diet in populations studied in different bariatric centres. Within the n-6 series PUFAs, of note is a significant decrease in arachidonic acid (ARA) content, which was not reported after RYGB [27, 29]. Obesity is associated with an increase in proinflammatory ARA metabolites [30]. The decrease in C-reactive protein (CRP) concentrations observed in our study is consistent with the improvement in serum inflammatory factors after bariatric surgery [31].

## Conclusions

We observed beneficial changes in some bioactive FAs, e.g. BCFAs, following OAGB in patients with morbid obesity but did not observe a restoration of PUFA levels nor the normalization of whole FA profiles. As OAGB is both a restrictive and malabsorptive procedure, the lack of complete normalization of FA profiles can be expected since the intake and absorption of dietary lipids is reduced. The continuous weight loss causes the mobilization of FAs in adipose tissue depots, which may lead to an increase in serum oleic acid. In the future, studies with a longer follow-up period might be useful to fully assess if FA levels fully restore after OAGB.
